# Cyanoacrylate Skin Surface Stripping and the 3S-Biokit Advent in Tropical Dermatology: A Look from Liège

**DOI:** 10.1155/2014/462634

**Published:** 2014-08-06

**Authors:** Gérald E. Piérard, Claudine Piérard-Franchimont, Philippe Paquet, Trinh Hermanns-Lê, Jean Radermacher, Philippe Delvenne

**Affiliations:** ^1^Laboratory of Skin Bioengineering and Imaging, Department of Clinical Sciences, University of Liège, 4000 Liège, Belgium; ^2^Department of Dermatopathology, Unilab Lg, University Hospital of Liège, 4000 Liège, Belgium; ^3^Department of Pathology, Unilab Lg, University Hospital of Liège, 4000 Liège, Belgium

## Abstract

In the dermatopathology field, some simple available laboratory tests require minimum equipment for establishing a diagnosis. Among them, the cyanoacrylate skin surface stripping (CSSS), formerly named skin surface biopsy or follicular biopsy, represents a convenient low cost procedure. It is a minimally invasive method collecting a continuous sheet of stratum corneum and horny follicular casts. In the vast majority of cases, it is painless and is unassociated with adverse events. CSSS can be performed in subjects of any age. The method has a number of applications in diagnostic dermatopathology and cosmetology, as well as in experimental dermatology settings. A series of derived analytic procedures include xerosis grading, comedometry, corneofungimetry, corneodynamics of stratum corneum renewal, corneomelametry, corneosurfametry, and corneoxenometry.

## 1. Background of Laboratory Aids in Tropical Dermatology

Most tropical dermatoses contracted by native residents, travelers, immigrants, and refugees are not life-threatening precluding basic diagnostic work-up. A series of simple laboratory tests are conveniently performed using minimum equipment for establishing a clinical diagnosis, clarifying a differential diagnosis or ruling out some specific alternatives. Practical advice on the diagnosis of tropical dermatoses is thus available in simple straightforward ways.

In most medical institutions diagnostic microbiology laboratories provide advice about adequate conditions for collecting, storing, and sending skin samples for establishing possible tropical bacterial, parasitic, or viral infections. During the early acute phase of infection, serum samples are possibly collected and stored in a freezer where available. Other distinct sampling procedures are available and have proven usefulness to practitioners facing a series of tropical dermatoses.

### 1.1. Potassium Hydroxide Preparation

A potassium hydroxide (KOH) preparation helps diagnosing most superficial fungal infections. Skin scales are gently scraped with the edge of a scalpel blade or of a glass slide onto a second glass slide. A drop of 10–20% KOH in water is added. Such collected stratum corneum (SC) material is covered with a coverslip and gently placed over a lighting match or candle until the preparation just begins to warm. The slide is examined at room temperature under a microscope after pulling down the condenser and partly closing the diaphragm. Fungal hyphae and spores (arthroconidia) are then conveniently disclosed.

### 1.2. Smear

In infectious lesions, cotton swabs are commonly used for obtaining suitable material for microscopic examination or culture. A smear is similarly performed by scraping the base of a vesicule with a scalpel blade and transferring the serum and cell debris to a glass slide. After heat or alcohol fixation, the specimen is stained in various ways depending on the searched disease. If adequate stains are not readily available, the fixed specimen is conveniently stored to be stained later or addressed to another facility.

Fecal smears from patients with acute and severe diarrhea are similarly sampled looking for ova, protozoans, and helminths. The presence of inflammatory cells possibly suggests shigellosis or amebiasis. Both of these conditions represent severe and potentially fatal infections. When necessary, stool samples are furthermore cultured for bacteria.

### 1.3. Sticky Transparent Tapes and Discs

Regular transparent self-adhesive tape is useful for collecting material and establishing the diagnosis of superficial dermatomycoses. A few centimetres of a regular sticky tape are stuck over an active scaly lesion. The material is firmly rubbed with the back of a fingernail. After removal, the tape harbours a close replica of the scaly lesion. Using such casual sticky tapes is not reliable for precise quantitative assessments because the adhesion to the SC is uncontrolled. An improved method has been called SACD for stripping with adhesive-coated discs [1].

The SACD sampling device is a crystal clear adhesive-coated disc (D-Squame, Cuderm Corporation, Dallas, TX, or Corneofix, C + K electronic, Cologne, Germany) providing the required stiffness and adhesiveness to uniformly sample a defined area of skin surface. After peeling off the protective seal, the disc is applied to the skin surface. When available, a gauge spring dynamometer ensures a controlled pressure onto the skin. Both the pressure and the application time of the disc influence the amount of collected SC. A short-time (about 5 s) application of the disc removes less SC than a long-time (about 1 h) application. Such difference results from occlusion increasing the SC hydration and decreasing the corneocyte cohesion.

### 1.4. Skin Snips

Skin snips are used for diagnosing onchocerciasis. They are performed with either a razor blade or a specific instrument for this procedure. The skin is first cleaned with an antiseptic solution; then it is pinched up between fingers or lifted with a needle. A small piece of skin, usually 2-3 mm in size, is quickly sliced off without anesthesia. Such a procedure is commonly well tolerated by the patient. The specimen is placed on a glass slide, covered with saline, and gently teased apart with forceps or needles. After placing a coverslip, the specimen is examined under low power magnification in a microscope looking for motile microfilariae.

### 1.5. The Matchstick Test

Demonstrating the presence of an apple-jelly nodule located in the upper dermis and covered by a stretched epidermis requires a regular wooden match sharpened to a point and placed vertically to the skin lesion. Light pressure with a finger causes the match to penetrate the epidermis and stand upright without support. If the lesion is deeply located in the dermis, the point of the match breaks under pressure. This test is positive in lupus vulgaris and lupoid leishmaniasis and negative in tuberculoid granulomata.

### 1.6. The Dental Broach Procedure

The smear method is frequently ineffective in ulcerated lesions and inadequate when a sample is expected from the depths of a dermal infection. Using a dental broach is preferable [2]. This instrument commonly used by dentists corresponds to a steel needle, the distal part being surrounded by metallic barbs of different sizes. The broach is easily inserted into the core of an infected skin area, producing a small puncture wound. After gently rotating the broach before removal, it becomes coated with tissue. The collected material as a stab procedure provides a smear for microbiologic examination.

In crusted lesions, it is usually possible to insert the broach from the side into the main body of the swelling, while avoiding any surface contamination. The technique is useful in diseases such as leishmaniasis, leprosy, anthrax, and yaws.

### 1.7. Conventional Biopsy

A biopsy is particularly useful for diagnosing most skin diseases. For the sampling procedure, a representative lesion is selected, cleaned thoroughly using an antiseptic solution, and infiltrated with a local anesthetic such as 1 or 2% xylocaine. When the lesion exhibits an annular configuration and appears to be enlarging, the advancing edge is the best location to biopsy. Otherwise, the central part of the lesion, unless it is necrotic, commonly yields appropriate information. The specimen is conveniently obtained with a punch biopsy trephine, or an elliptic section is performed using a scalpel. It is often important to collect the skin sample down to the hypodermis. Sutures are commonly used for closing the biopsy site when its diameter is larger than 4 mm. The specimen is conveniently splinted into several pieces when some specific procedures are requested such as cultures or molecular biology. The specimen for histopathologic examination should be placed in 10% buffered formalin or any other suitable fixative. When dermatopathology facilities are not readily available, the specimen is conveniently processed at a later time within a couple of weeks.

Detailed accounts of more sophisticated diagnostic procedures are available in centers of excellence often located in the Western world. They are of little practical value to physicians in contact with patients living in the jungle, in small hospitals of the countryside, and in area where poverty of the population makes it unlikely to be able to afford some recent expensive investigations.

### 1.8. Cyanoacrylate Skin Surface Stripping

The cyanoacrylate skin surface stripping (CSSS) will be particularly described in depth in this review paper. CSSS entails the specific collection of the upper portion of the SC. Restricted economic conditions in developing countries preclude using sophisticated diagnostic procedures. Therefore, time-tested inexpensive laboratory methods such as CSSS are stressed. Fortunately, in the great majority of dermatoses, a reasonably accurate diagnosis can be reached clinically and occasionally in combination with CSSS. In such a process, the key point is the accurate understanding of the SC aspect and biology.

## 2. Stratum Corneum Structure

The SC consists of the ordered association of dead corneocytes exerting a prominent barrier function leading to partial protection from ultraviolet (UV) light, microorganisms, and various toxic xenobiotics. In addition, the SC protects against uncontrolled loss of water, electrolytes, and macromolecules from the skin. Thus, it shields in part the deeper living tissues from various environmental threats. The SC further acts as a crucial biosensor signalling the underlying living epidermal layers for possible responses to external stresses. Despite minimal metabolic activity, the SC represents a highly dynamic structure resulting from the continuous corneocyte renewal. Such a process ideally keeps a steady state in the SC structure and thickness. Of note, corneocytes are structurally and biochemically heterogeneous at the surface of the SC [3].

Over most of the body surface, the SC is typically composed of fifteen or so layers of flattened corneocytes. These cells at the skin surface are about 1 *μ*m thick, and their mean area reaches approximately 1000 *μ*m^2^. The corneocyte area is influenced by the anatomic location and various conditions including chemical irritation and UV action modulating the epidermal renewal. In addition, the average corneocyte size is assumed to increase with aging. This feature is probably associated with a prolonged corneocyte transit time in their way throughout the SC.

The water holding capacity of the SC keeps its surface soft and smooth. Indeed, some of the SC molecular components bind water and prevent its evaporation from the skin surface. These compounds constitute the so-called natural moisturizing factor (NMF), corresponding to a mixture of small water-soluble molecules including amino acids, lactate and urea, intercellular lipids, sebum, and specific protein components of corneocytes. Abnormalities in the relative amount of these molecular components commonly lead to a harsh and stiff SC and eventually to fine cracking and fissuring.

Knowledge about the fine SC structure is crucial in the understanding of many respects of dermatology. In addition, dermocosmetic science similarly benefits from advances in the field, particularly when dealing with age-related xerosis and effects of surfactants, emollients, and squamolytic agents. In some physiopathological instances, the SC homeostasis is altered. The SC structure is indeed influenced by diverse and repeat external threats. In addition, the genetic background, the nutritional status, and some physical agents, as well as drugs, cosmetics, toiletries, and many other chemical xenobiotics represent additional modulators of the SC structure. In short, the SC becomes the repository of many biologic events that previously influenced the activity of the underlying keratinocytes.

## 3. Cyanoacrylate Skin Surface and Follicular Stripping

CSSS came into existence when high bond glues became available to be applied to the SC [4]. After its initial description, it was soon applied for diagnostic purposes in a set of skin disorders [5]. Sampling on polyethylene strips was a primordial improvement leading to the subsequent expansion of the method [6]. Indeed, such supple transparent strip is preferable to glass slide for two main reasons. It is indeed easier to get a close modelling of any curved body area. In addition, the adhesion of the SC to the polyethylene strip is so strong that corneocytes are not lost during the subsequent laboratory procedures.

Performing a CSSS consists of depositing a drop of cyanoacrylate liquid adhesive onto a supple transparent polyethylene sheet, 175 *μ*m thick, cut to about a regular coverslip size (1.5∗6 cm). The sampling material is currently available as a kit (3S-Biokit, C+K electronic, Cologne, Germany). The material is pressed firmly onto the target site of the skin. After 15–30 s, a thin sheet of SC is conveniently harvested. The cleavage level is exclusively located inside the SC [7, 8]. Of note, oozing and eroded lesions cannot be sampled using CSSS.

CSSS can be performed from any body region, with two main limitations. Indeed, harvesting CSSS from a hairy area is often painful due to pulling out hairs. The CSSS quality is then inadequate owing to the erratic contact of the cyanoacrylate glue with the SC. It is therefore advisable to shave such areas before any CSSS sampling. Another problem results from the natural strong intercorneocyte cohesion on the palms and soles. It is commonly stronger than the glue bond. This impairs the collection of a uniform thin layer of corneocytes. However, a CSSS sampling on these sites is possible in certain physiopathologic conditions in which the SC cohesiveness is compromised.

When vellus hairs are dispersed on the examined skin site, they are harvested by CSSS. In addition, the CSSS collects follicular casts corresponding to the horny material present at the opening of pilosebaceous follicles at the skin surface. In the past, this sampling procedure has been specifically called follicular biopsy [9]. By this way, it is possible to assess the density of hair follicles per surface area and to observe the presence of follicular hyperkeratosis (kerosis), as well as comedones,* trichostasis spinulosa*, intrafollicular bacteria, and mites [7–11]. In some instances, other hair follicle structures such as hair bulbs and follicular sheaths are visualized on CSSS. Skin pores distinctly corresponding to follicular or sudoral openings at the skin surface are conveniently explored using CSSS [12].

## 4. Overall Aspect of Normal Skin on CSSS

**Figure 1:**
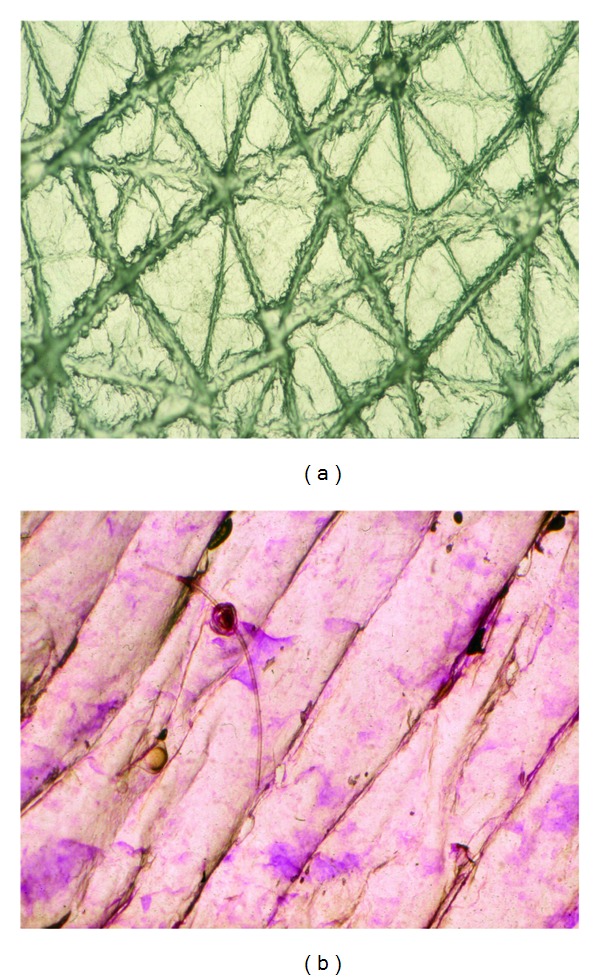
CSSS from the forearm. (a) Young subject showing the criss-cross pattern of shallow lines. (b) Older individual with parallel orientation of shallow lines.

CSSS from healthy skin reveals a regular network of high-peaked crests corresponding to discrete skin surface creases called the first-, second-, and third-hollow order lines [3–8, 13]. Their patterns of distribution are typical for specific body regions. The first-order lines correspond to grooves in the latticework papillary relief at the dermoepidermal interface [14, 15]. In young individuals, intersections of the first- and second-order lines demarcate polyhedral shaped SC plateaus (Figure 1(a)). On stretching the skin surface a realignment of these lines occurs in part. With aging, this network progressively loses its original configuration, spontaneously aligning itself preferentially along the skin tension lines (Figure 1(b)). The process ends with vanishing of the shallow skin surface creases [8]. It is therefore possible to assess by that indirect way the texture of the superficial dermis on CSSS. As a result, dermal aging, corticosteroid-induced atrophy (dermatoporosis), striae distensae, sclerosis, scars, and many other connective tissue changes are conveniently observed noninvasively using CSSS. Such morphologic assessment of the skin microrelief is possibly quantified using computerized image analysis and profilometry methods [7, 8].

**Figure 2:**
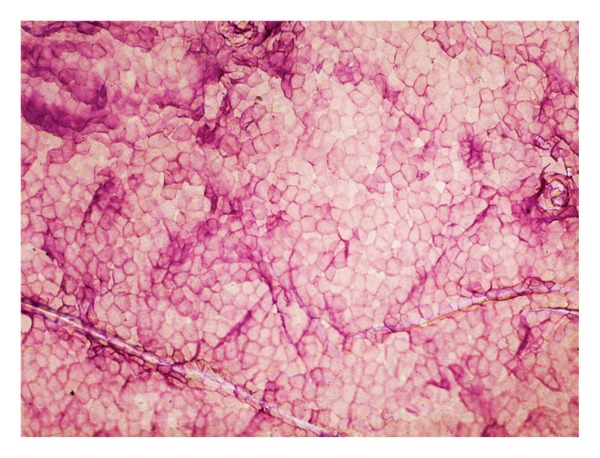
Regular paving of corneocytes.

Cytologic aspects of corneocytes are hardly visible on unstained CSSS by microscopic dyes [6, 8, 16]. Several histochemical stains are useful. A mixture of toluidine blue and basic fuchsin (TBBF) in 30% ethanol is a simple and convenient one easy to handle in an office setting [7, 8]. The normal SC exhibits a uniform cohesive pattern of adjacent corneocytes (Figure 2). Each corneocyte is typically anucleated and contains a water-insoluble protein complex made predominantly of a highly organized keratin microfibrillar matrix.

**Figure 3:**
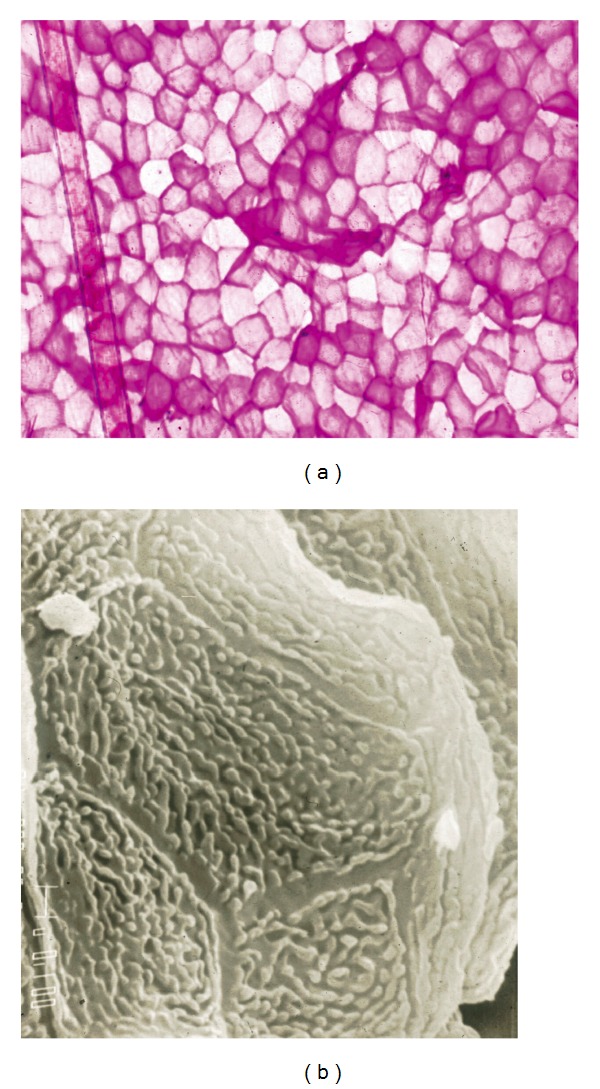
Stratum corneum maturation. (a) Heterogeneity in corneocyte maturation with mature clear cells and immature deeply stained cells. (b) Immature corneocyte with discrete uniform protrusions (scanning electron microscopy).

Corneocytes are encapsulated in a protein and lipid-enriched shell. The cell boundaries are clearly stained by a thin polyhedral TBBF rim. The cornified cell envelope exhibits differences in maturation among corneocytes. Basically, two distinct types of cornified cell envelopes are distinguished, namely, the fragile immature envelopes and the rigid mature ones [3, 17]. The former type is recognized on CSSS by a deep staining with TBBF (Figure 3(a)). By scanning electron microscopy, immature corneocytes often exhibit a paving of small protrusions of similar sizes (Figure 3(b)).

**Figure 4:**
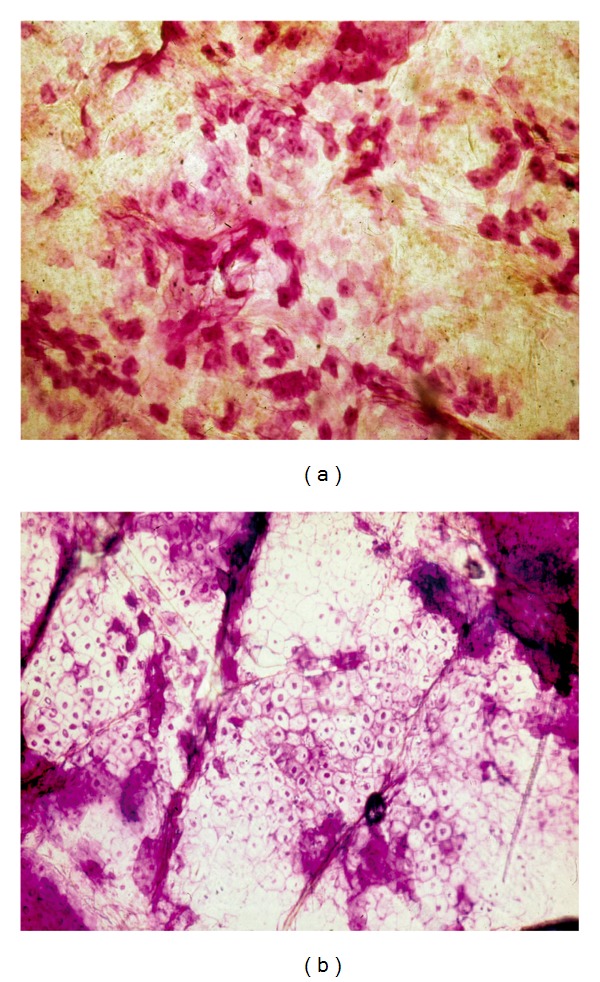
Parakeratotic cells. (a) Dispersed pattern. (b) Cohesive pattern in seborrheic dermatitis.

On healthy skin, parakeratotic cells are rare, and those present are not clustered (Figure 4(a)). By contrast, clumps of parakeratotic cells usually suggest a pathologic process (Figure 4(b)). They are recognized by the presence of a nucleus central to the polyhedral cell.

Lipid staining such as the Red Nile stain conveniently reveals the sebum-enriched follicular pores and follicular casts. Any histochemical positivity at the follicular sites corresponds to two distinct conditions [12]. First, the visualized sebum reflects the direct lipid production by each single follicle. Second, the lipids were produced and poured out by other follicles in their vicinity, run at the skin surface, and finally collected in the declivity of other follicular openings.

The biocene of resident bacteria and other saprophytic microorganisms is typically confined to the skin surface and the appendages. It remains largely encased inside the cyanoacrylate bond during sampling. Thus, it is not accessible to staining procedures and it remains invisible at the microscopic examination. Therefore, the surface microflora is not adequately disclosed on CSSS. By contrast, samples of microorganisms entrapped inside follicular casts are easily collected distinctly from the skin surface microflora by scraping out the horny spiky structures appending to the CSSS. Viability of the intrafollicular bacteria is conveniently assessed using the combination of vital stains and flow cytometry [18].

Sensitive skin corresponds to a condition characterized by a reduced cutaneous tolerance to a variety of environmental factors (cold, heat, wind, wool, topical products, etc.). Clinical manifestations consist mainly of subjective symptoms linked to sensory irritation including discomfort, itching, stinging, and burning sensations. There are no specific signs discernable on CSSS except occasional discrete xerosis and parakeratosis.

## 5. Diagnostic CSSS in Inflammatory Dermatoses

The regular SC exhibits both protective biologic efficiency and esthetic qualities. It is rarely considered as a structure causing serious disablement when diseased. However, a large amount of the clinical work load of dermatologists deals with disorders associated with scaling and/or SC thickening. Psoriasis, ichthyoses, eczematous dermatoses, and the panel of xeroses represent typical examples of such disturbances. These common conditions are characterized by abnormal epidermal maturation and scaling. Relatively few studies were performed on the clinical consequences of the abnormal SC maturation despite their frequency and the problems they cause in most populations. CSSS helps exploring these disorders.

Obviously, the diagnostic indications for CSSS are restricted to disorders exhibiting some SC involvement. The most common conditions diagnosed by CSSS were previously explored [5–8, 19–22] and are summarised in Table 1. Learning how to interpret the microscopic findings on CSSS requires minimal experience that is well in the expertise of clinical practitioners.

### 5.1. Infectious Dermatoses

**Figure 5:**
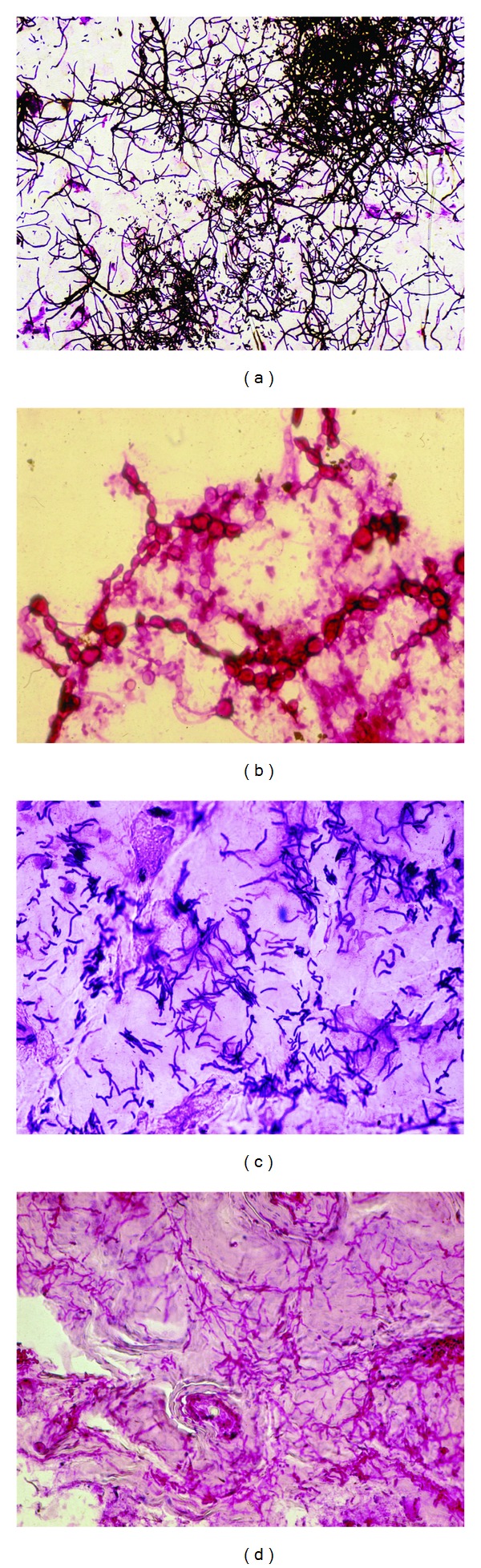
Fungal invasions. (a) Dermatophyte infection. (b)* Candida* sp. infection. (c)* Malassezia* sp. in pityriasis versicolor. (d) Tinea capitis.

Straightforward diagnoses are reached by CSSS in a number of superficial infectious and parasitic dermatoses [5–8, 19, 21, 22]. Microscopic examination, possibly combined with fungal culture, is easily carried out for identifying such types of lesions. Infectious agents that are visible on CSSS are not those adhering to the skin surface (see above), but rather those invading the SC. The PAS and Gomori-Grocott stains are well suited for revealing hyphae, arthroconidia, and yeasts on CSSS. Fungi of the yeast and dermatophyte classes show their typical morphology (Figures 5(a), 5(b), and 5(c)). They form clusters and networks of globular and filamentous structures. In some instances, only ghost-like hyphae are seen. In addition, it is possible to apply vital stains such as Neutral Red in order to distinguish living fungi among corneocytes and dead fungi following or not antifungal treatments. The proportion of dead/alive fungi is related to the efficacy of the antifungal therapy at the time of the CSSS sampling. Such information is not accessible by culture that only reveals the presence of surviving and growing fungi.

Tinea capitis is a peculiar fungal infection of the scalp that is conveniently diagnosed using CSSS (Figure 5(d)). This method selectively possibly removes infected hair shafts that are more loosely attached inside the follicle than intact hairs. Infected hairs appear short, stubby, and swollen in comparison to the longer, smooth, and thinner healthy hairs. On high-power magnification, hyphae are seen within the hair shaft in endothrix tinea or in a cuff outside the hair shaft in ectothrix tinea.

### 5.2. Parasitic Dermatoses

**Figure 6:**
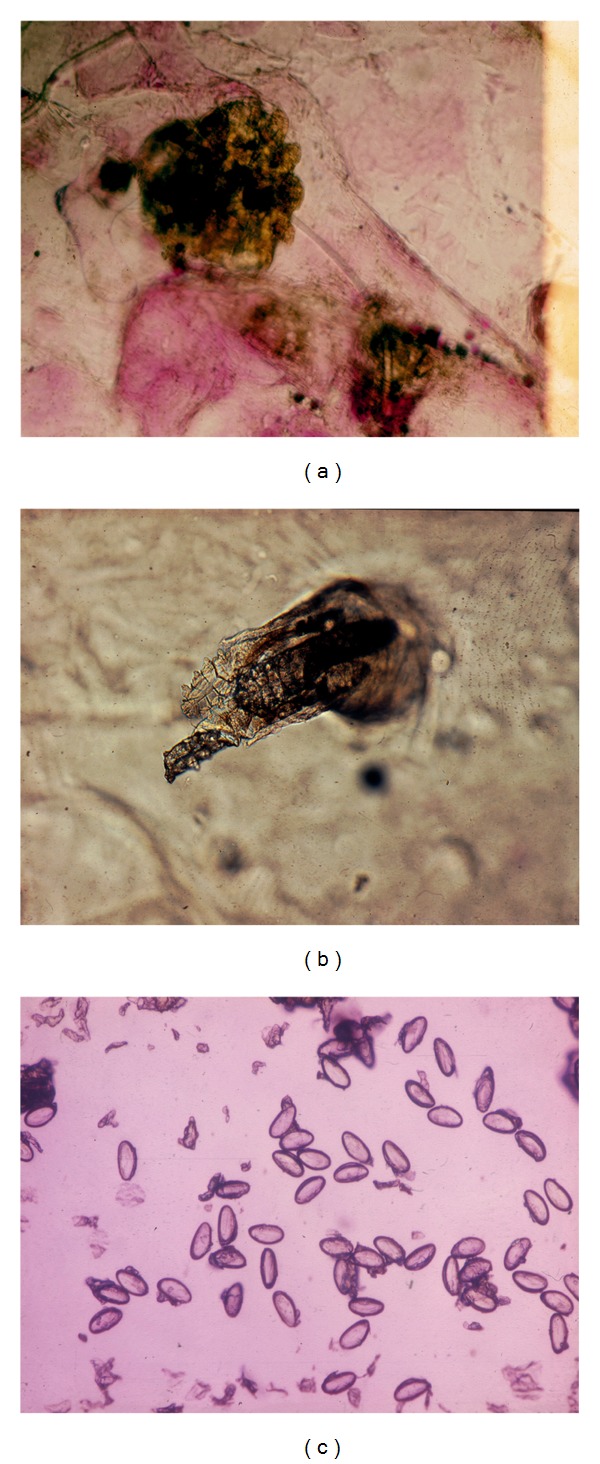
Superficial parasitoses. (a) Acarus scabiei. (b)* Demodex folliculorum*. (c) Oxyuriasis.

In the group of skin parasitic dermatoses, scabies commonly represents a problem at the CSSS sampling [5, 7]. In fact, the diagnosis is established only when the mite, its eggs, or dejecta are present in the examined CSSS. In general, duplicate or serial CSSS samplings should be harvested from any suspected scabies burrow. The first one intends to remove the burrow roof. The next ones are probably better suited for harvesting the parasite (Figure 6(a)). Any sampling collected outside such parasitic lesion, for instance, from unspecific prurigo, is inadequate because the diagnosis will merely suggest the presence of spongiotic dermatitis [7, 8, 22].

Demodex mites (Figure 6(b)) are conveniently recognized in follicular casts [7, 8, 23, 24]. They are highlighted by the Fite stain [7]. Their numerical density on facial skin is probably related to the severity of demodicidosis.

Oxyure ova are clearly identified on CSSS (Figure 6(c)).

### 5.3. Foreign Body Particles

Some exogenous materials adhering to the skin are conveniently collected by CSSS. An example is given by plant trichomes causing trichomatoses. Polarized light examination markedly increases the visibility of trichomes in the SC [25]. Fiberglass is detected as well.

### 5.4. Spongiotic and Parakeratotic Dermatoses

**Figure 7:**
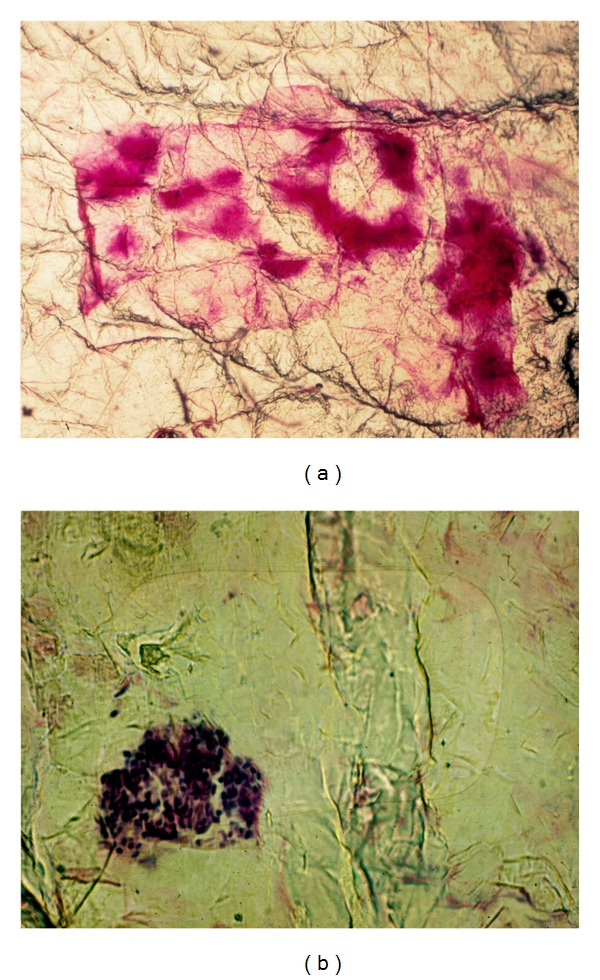
Spongiotic-parakeratotic disorders. (a) Pityriasis rosea with small serum deposits. (b) Clump of neutrophils in active psoriasis.

Noninfectious erythematosquamous disorders recognizable on CSSS include a series of spongiotic and parakeratotic dermatoses and xeroses as well [7, 8, 21, 22]. Spongiotic dermatoses correspond to superficial inflammatory reactions responsible for spongiosis, microvesiculation, and serosity leakage inside the SC (Figure 7(a)). Spotty parakeratosis is commonly present as well. Contact dermatitis, atopic dermatitis, and pityriasis rosea typically belong to this class of diseases [7].

Parakeratotic dermatoses encompass diseases such as reactions, chronic eczema, and stable psoriasis. Parakeratotic cells are commonly clustered in sheets or in thicker collections (Figure 4(b)). Contrasting with the topography of uninvolved skin, the surface of psoriatic lesions and actinic porokeratosis demonstrates alterations in the regular geometric pattern of the shallow skin lines. In addition, actinic porokeratosis is revealed by a rim of cornoid lamellation. Seborrheic dermatitis comes within this category of parakeratotic disorders with possible presence of* Malassezia* yeasts. In active psoriasis, clusters of neutrophils (Figure 7(b)) are recognized on top of parakeratotic clumps.

## 6. Diagnostic CSSS in Cutaneous Neoplasms

**Figure 8:**
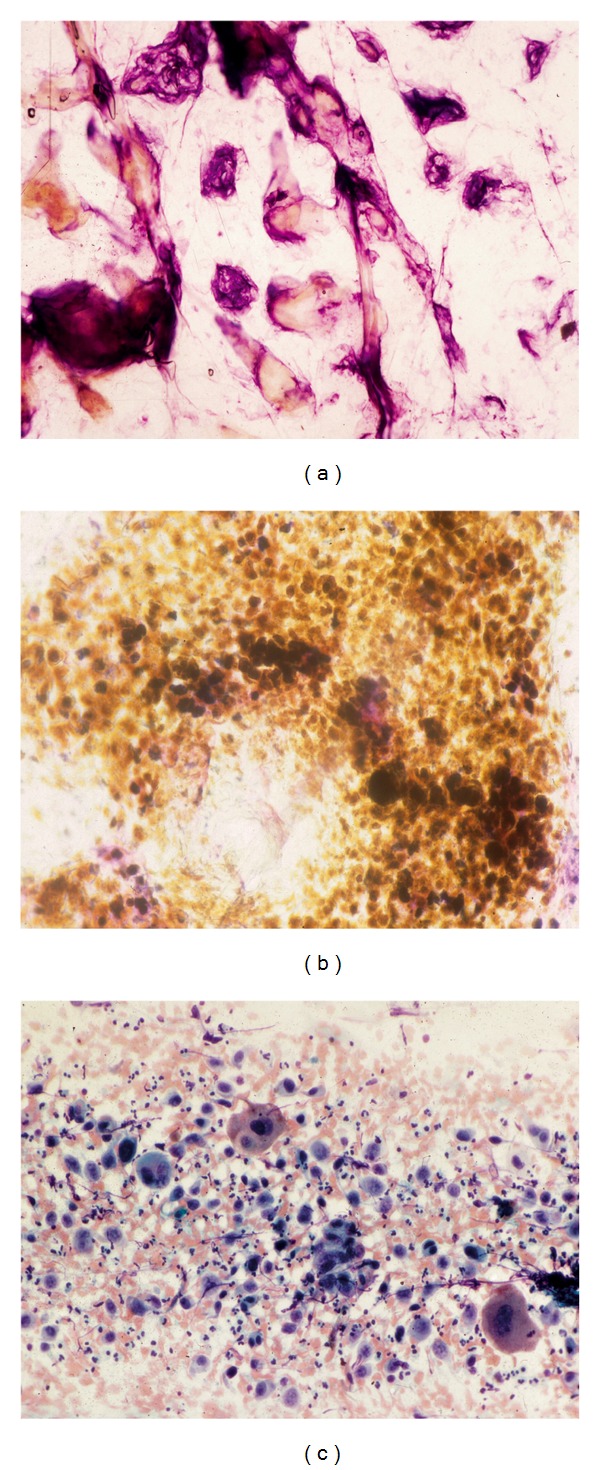
Epithelial neoplasms. (a) Seborrheic keratosis. (b) Malignant melanoma. (c) Carcinoma of the genital area.

Some epithelial neoplasms display characteristic presentations on CSSS [7, 8]. A sharp circumscription by a normal-looking surrounding skin and uniformity of the changes in the texture of the SC are commonly abutted to benign neoplasms. Seborrheic keratoses are characterized by spotty lenticular foci of soft hyperkeratosis (Figure 8(a)). Enlargement of shallow furrows filled in by hyperkeratosis is typically present. CSSS samples of actinic keratosis commonly exhibit uneven thickness with interfollicular parakeratosis and xerosis. Verrucous surfaces overlying melanocytic nevi and dermatofibromas are less pathognomonic.

In CSSS taken from pigmented skin neoplasms, melanin deposits are usually present inside corneocytes and/or atypical melanocytes. Melanin located only inside corneocytes is commonly a feature of benign neoplasms, including juvenile and solar lentigines. Presence of atypical melanocytes in the SC strongly suggests a possible malignant melanoma (Figure 8(b)), but also, in rare instances, a benign melanoacanthoma [7, 8, 26, 27]. Thus, CSSS proves to be sensitive and specific in the distinction between malignant melanoma and benign melanocytic tumors such as common melanocytic nevi, dysplastic nevi, and pigmented seborrheic keratoses [26]. For research purposes, karyometry of neoplastic melanocytes is possibly performed on most CSSS [27].

Most basal cell carcinomas and squamous cell carcinomas do not usually exhibit any specific or suggestive features. However, carcinomas of the genital area occasionally exhibit atypical keratinocytes (Figure 8(c)).

## 7. CSSS Analytical Assessments

Some analytical measurements are conveniently performed on CSSS. They commonly rely on the combination of optical properties of the samples, colorimetry, and morphometry-based image analysis. Some aspects of skin disease severity and therapeutic improvement are conveniently assessed on CSSS showing typical features in the SC.

### 7.1. Xerosis Grading

Xeroses correspond to various forms of predominantly orthokeratotic hyperkeratosis [28]. Such SC alterations correspond to the so-called “dry skin” for the laity, although the condition recalls some presentations of the ichthyosis group [7, 8, 21, 28–30]. Clearly, several grades of orthokeratotic hyperkeratosis are detected on CSSS [7]. Type 0 refers to the absence of hyperkeratosis, except for some discrete focal accumulations of corneocytes inside the first-order lines of the skin. Type 1a corresponds to a continuous linear hyperkeratosis of the first-order lines. Type 1b is characterized by hyperkeratosis predominating at adnexal openings at either hair follicles or acrosyringia. Type 2 corresponds to focal hyperkeratosis of the skin surface plateaus representing less than 30% of the total area of the sampling. Type 3 resembles type 2, but with an altered area over 30% of the CSSS. Type 4 is defined by homogeneous diffuse hyperkeratosis with persistence of the first-order lines. Type 5a resembles type 4, but with loss of recognizable shallow lines. Type 5b corresponds to the most heterogeneous and diffuse hyperkeratosis with loss or marked remodelling of the shallow line network. In ichthyoses, cracks, large fissures, clefts, and splits break up the surface topography. The excessive SC thickening is seen as multilayered corneocytes stacked on top of each other.

### 7.2. Corneofungimetry

In superficial dermatomycoses, fungal cells are readily visible on CSSS. In experimental settings, some assessments of dermatomycosis severity and therapeutic activity are conveniently performed on CSSS using morphometry and computerized image analysis.

In particular, some quantifications of dermatomycosis severity and antifungal activity are performed using corneofungimetry on CSSS [31, 32]. Microscopic fungi can be cultured in vitro using corneocytes as a growth substrate, particularly on CSSS. Quantifications of the restricted fungal growth after application of antifungals in experimental dermatomycoses are performed. The oral or topical antifungals are administered to healthy volunteers for a given period of time, usually a couple of days [31]. CSSS are sampled afterward. A controlled amount of fungal cells collected from a primary culture is deposited onto the CSSS supposedly impregnated with the test antifungal. After a given period of time, usually a dozen days of culture on CSSS in a clean and controlled environment, the CSSS samples are stained for revealing the presence of fungi. Computerized image analysis is used to fine-tune the quantification of the mycelium growing on CSSS. The comparison with controlled untreated CSSS allows deriving the inhibition percentage of the fungal growth.

Corneofungimetry has several advantages over conventional in vitro evaluation of antifungals: (a) the treatment is applied in vivo in conditions normally encountered by patient, (b) the initial fungal load is controlled, (c) the growth medium is only composed of corneocytes without any other chemical compounds, and (d) any influence of corneocytes including the natural antimicrobial peptides is preserved.

### 7.3. Corneomelametry

Melanin is identified in normal corneocytes of phototype V and VI individuals. It is important to distinguish melanin-laden anucleated corneocytes from neoplastic dendritic nucleated melanocytes after their migration inside the SC in malignant melanomas [26]. The dusty melanin load is typically revealed using argentaffin stain procedures. The relative darkness of these CSSS is conveniently assessed using corneomelametry [33–35]. Such a method consists of measuring the reduction of light transmission through the CSSS using a photodensitometer device designed for photomicroscopy.

### 7.4. Corneodynamics

Corneodynamics refers to the dynamics of SC renewal. It is conveniently assessed using CSSS collected about a dozen days after topical application of a fluorescent or a dye deeply staining the SC. The more the SC renewal is rapid, the less the stain remains present in the short term on the CSSS.

**Figure 9:**
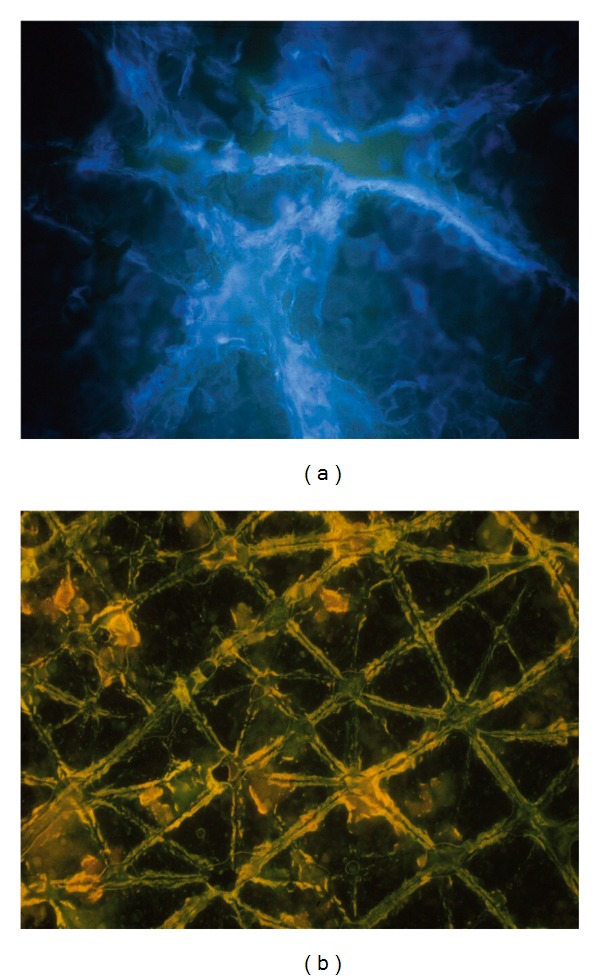
Persisting fluorescence of the stratum corneum. (a) Uneven fluorescence. (b) Persisting fluorescence of the shallow lines.

Dansyl chloride is a time-honored fluorescent compound for the SC. For years, the test relied on daily assessment of the decline in the clinical fluorescence [36]. The rate of SC renewal was determined by the duration of the fluorescence persistence. However, this clinical test proved to be difficult to interpret because it was not easy to clinically evaluate with precision the moment of fluorescence loss due to the uneven fade-out of fluorescence. The CSSS method is a variant procedure performed at a predetermined time after dansyl chloride application. The fluorescence pattern is quantified with precision (Figure 9(a)) using image analysis under fluorescence microscopy [37]. In most instances, the shallow skin lines represent typical sites for the largest residual fluorescence (Figure 9(b)).

Fluorescence fading is assessed in vivo after application of topical products and interpreted as an effect on the keratinocyte renewal [38]. However, this procedure possibly represents a pitfall when the test product removes dansyl chloride from the SC [39]. The adequate procedure should begin with the application of the test product for at least a dozen days. In a second step dansyl chloride is applied without any further applications of the test product. Such a procedure allows disclosing any boosting effect on the epidermis without introducing the risk for artefactual dansyl chloride extraction.

A risk of allergy and systemic resorption of dansyl chloride is possible. Hence, there is some limitation for its use, particularly in subjects involved in a series of similar tests. Dihydroxyacetone was offered as a surrogate SC marker [40].

### 7.5. Corneosurfametry and Corneoxenometry

**Figure 10:**
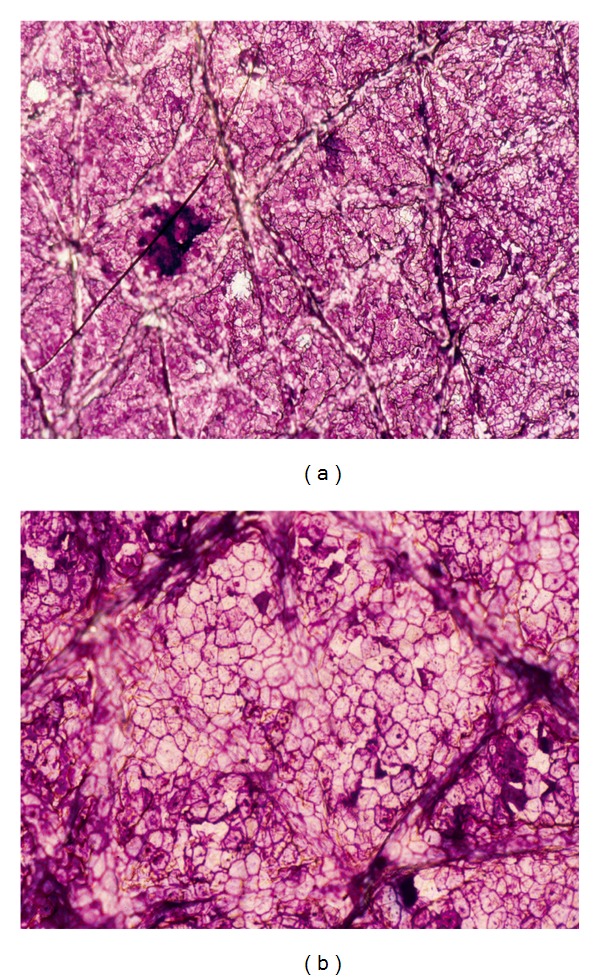
Corneosurfametry. (a) Dense positive staining throughout the CSSS. (b) Uneven surfactant reactivity of corneocytes.

The impact of various chemical xenobiotics on the SC is conveniently assessed on CSSS. Corneosurfametry (CSM) refers to the effects of surfactants and wash solutions. CSSS are initially harvested from healthy skin of volunteers [41]. A solution of the test product is sprayed on each CSSS. The material is placed in plastic trays covered by a lid. After a given time of incubation at controlled temperature, the samples are thoroughly rinsed in tap water, dried, and stained for 3 min in TBBF solution. Samples are then profusely rinsed with water and dried prior to color determination using reflectance colorimetry. Indeed, surfactants remove lipids and denaturate corneocyte proteins, thus revealing sites available for staining deposition and transient corneocyte edema as well [42]. A combined dotted and rimmed pattern is visible on corneocytes under microscopic examination (Figures 10(a) and 10(b)).

Using quantitative reflectance colorimetry, mean luminancy (L*) and Chroma C* are calculated from measurements made at three sites on each sample placed on a white reference tile. Mild surfactants with little damaging effect on corneocytes give a combination of high L* values and low Chroma C* values. The L* value decreases and Chroma C* increases in concert with the irritancy potential of the product. The differences between L* and Chroma C* values for each sample give colorimetric indices of mildness (CIM). The CSM index (CSMI) of the test product corresponds to the color difference between water-treated control samples and those exposed to the test product. CSMI is conveniently calculated as follows: CSMI = [(ΔL*)^2^ + (ΔC*)^2^]^0.5^.

Microwave CSM is a more rapid procedure. CSSS are immersed in a flask containing the test surfactant solution. Samples are then placed in a microwave oven with a 500 mL water load. Microwave CSM is typically run at 750 W for 30 s [43]. The next steps are identical to the standard CSM procedure.

Responsive CSM is a variant of the method where skin is pretreated before CSSS sampling [44]. The method is based on repeat subclinical injuries by surfactants monitored in a controlled forearm immersion test. At completion of the in vivo procedure, CSSS are harvested for a regular or microwave CSM bioassay using the same surfactant as in the initial in vivo procedure. Preconditioning the skin by this way increases CSM sensitivity to discriminate among mild surfactants.

Shielded CSM is used for testing skin protective products (SPP). SPP claiming a barrier effect is expected to act as shields against noxious agents. In shielded CSM, the CSSS are first covered by the test SPP before performing regular CSM using a reference surfactant. Comparative screenings of SPP are conveniently performed using shielded CSM without exposing volunteers to hazards linked to in vivo testing.

Animal CSM is performed in a way similar to human CSM [45]. The method is available for safety testing of cleansing products specifically designed for some animal species. In addition, interspecies differences in surfactant reactivity of the skin are conveniently assessed.

The corneoxenometry (CXM) bioassay is used for testing any chemical xenobiotic other than surfactants. The basic procedure is similar to CSM and its variants. One main indication is found in the field of skin irritation while avoiding in vivo hazards [46, 47]. Skin protection creams are conveniently tested using CXM [48]. Another indication concerns the comparative assessment of penetration enhancers commonly used in topical formulations [49].

### 7.6. Comedometry

**Figure 11:**
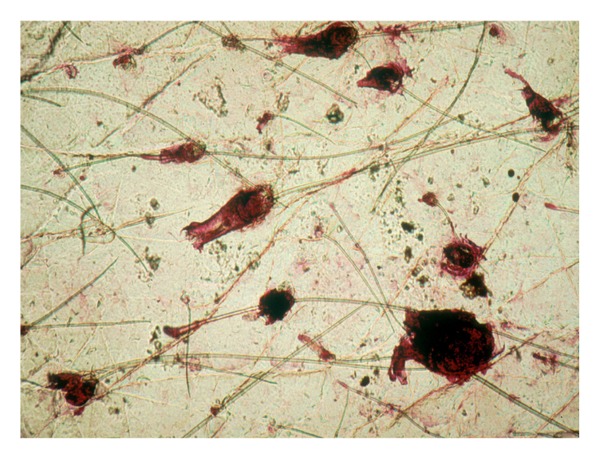
Comedometry-microcomedones of various sizes in acne.

Comedometry allows computerized quantification of the number and size of follicular casts present on CSSS. The numerical density of follicles is related to the body site, and for each site the interindividual variation is small. This method finds application in the comedogenesis and comedolysis-related disorders and treatments [18, 50–53]. Comedometry on human skin appears more relevant than animal (rabbit ear) models of comedogenesis. In similar testing conditions, large interindividual differences appear in the number of horny follicular casts. When an exogenous comedogenic factor is involved, the vast majority of the follicles are similarly affected. By contrast, endogenous comedogenic factors (androgens, etc.) typically affect at variable extent a minority of hair follicles (Figure 11). The sensitivity of the method is such that microcomedolysis is possibly objectivated after a few days or weeks of adequate treatment [54].

Sebum-sensitive foils (SSF) corresponding to Sebutape (Cuderm corp, Dallas, USA) or Sebufix (C + K electronic, Cologne, Germany) are conveniently used for assessing the sebum output at the skin surface. It is possible to combine such method with CSSS [55]. In a first step, SSF is applied to the skin for a couple of minutes. The outlines of the foil are ink-marked on the SC. In a second step following removal of the SSF, CSSS is collected from the very same skin site. The ink mark is visible on this sampling. The CSSS and the foil are then exactly superposed using the ink mark as an adjusting mark. The dual SSF-CSSS samplings are examined under the microscope and submitted to image analysis considering distinctly the darker horny follicular casts and the clear transparent sebum spots. Correlations are possibly established between the follicular pore sizes, microcomedones, and the follicular sebum output [55].

## 8. Conclusions

Beyond conventional biopsies and cytology of exudates, touchprints, and scrapings, CSSS provide useful information in the field of clinical dermatology, dermatopathology, and skin pharmacology. This simple, low cost, and minimally invasive method allows the clinician to avoid invasive procedures within limits of well-defined indications. Less than 3 min is required between SC sampling and its microscopic examination. There are obvious features and subtle characteristics discernible in the structure of the SC for establishing diagnosis in a variety of skin diseases including tropical dermatoses. It is important to stress that no single criterion should usually be relied upon for a definitive diagnosis on CSSS. Rather a constellation of clues should be sought. Quantifications are made possible on CSSS using computer-assisted image analysis.

## Figures and Tables

**Table 1:** Indications for surface biopsy.

(1) Superficial infections Molluscum contagiosum Bacterial diseases (impetigo, erythrasma, etc.) Dermatophytosis Candidosis Pityriasis versicolor	
(2) Superficial parasitoses Scabies Demodicidosis Oxyuriasis	
(3) Xeroses and erythematosquamous, spongiotic and parakeratotic dermatitides Xerosis—kerosis—ichthyosis Eczema—contact dermatitis Atopic dermatitis Pityriasis rosea Id reaction Psoriasis Seborrheic dermatitis	
(4) Tumors Malignant melanoma Melanocytic nevus Dysplastic nevus Seborrheic keratosis	
